# Quantification of cholesterol incorporation in giant unilamellar vesicles produced by a modified cDICE method

**DOI:** 10.1039/d5sm01124h

**Published:** 2026-03-05

**Authors:** Marcos Arribas Perez, Gijsje H. Koenderink

**Affiliations:** a Department of Bionanoscience, Kavli Institute of Nanoscience, Delft University of Technology 2629 HZ Delft The Netherlands g.h.koenderink@tudelft.nl

## Abstract

Cholesterol is an essential component of eukaryotic cell membranes, influencing membrane packing, fluidity, and domain formation. Replicating these properties in model membranes is critical for reconstitution studies, but common emulsion-based methods for producing giant unilamellar vesicles (GUVs) fail to incorporate cholesterol efficiently. Here, we use methyl-β-cyclodextrin–cholesterol (MβCD–CL) complexes to deliver cholesterol into GUVs produced by the emulsion droplet interface crossing encapsulation (eDICE) method and demonstrate a convenient way to quantify the degree of cholesterol incorporation using fluorescent membrane biosensors. Spectral imaging of NR12A as well as fluorescence lifetime imaging of Flipper-TR revealed dose-dependent increases in cholesterol content for DOPC GUVs upon MβCD–CL addition, consistent with increased membrane order. By calibrating these effects against GUVs with defined cholesterol contents prepared *via* gel-assisted swelling, we found that the cholesterol content of eDICE vesicles can be increased to at least 40 mol%. Binary mixtures of DOPC with saturated lipids (DMPC and PC (18 : 0–14 : 0)) showed a similar trend as pure DOPC GUVs. Interestingly, we could trigger liquid-ordered domain formation by adding cholesterol to DOPC : DMPC vesicles. Our findings provide a quantitative and non-disruptive method to modulate and assess cholesterol content in emulsion-based GUVs, advancing their use in bottom-up synthetic biology and membrane biophysics.

## Abstract 2

MβCD-mediated cholesterol incorporation into emulsion-based GUVs quantified *in situ* using environment-sensitive fluorescent dyes.

## Graphical abstract

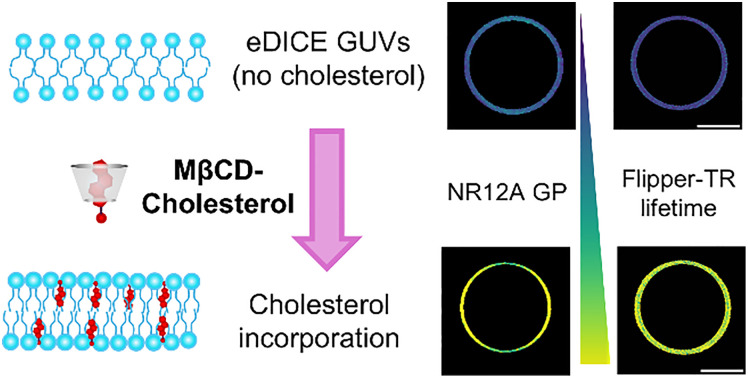

## 1. Introduction

Cell membranes are dynamic structures that, far from being simple barriers, play key roles in a wide range of cellular activities, including compartmentalization, signal transduction, membrane trafficking, and cell shape regulation.^1^ They are composed of a diverse mixture of lipids, proteins, and glycolipids, organized in a highly regulated and heterogeneous manner.^1,2^ The lipid bilayer itself contains hundreds of distinct lipid species, which are asymmetrically distributed across the two leaflets and contribute to the structural and functional complexity of cell membranes.^3^ A key component of eukaryotic plasma membranes is cholesterol, which constitutes between 10 and 30% of the total lipid content.^1^ Cholesterol critically influences membrane characteristics including thickness, rigidity, packing, and permeability.^4^

To study the biophysical properties of cell membranes in a controlled manner, giant unilamellar vesicles (GUVs) have become a widely used biomimetic model system. These cell-sized lipid vesicles mimic the architecture of biological membranes and allow control over the membrane physicochemical properties as well as the reconstitution of minimal cellular processes under controlled environments.^5,6^ Achieving cholesterol incorporation is essential for generating membrane models that faithfully replicate the composition and physical properties of cellular membranes. Furthermore, in GUVs made of mixtures with saturated and unsaturated lipids, cholesterol supports the formation of coexisting liquid-ordered (L_o_) and liquid-disordered (L_d_) domains.^7^ These phase-separated membranes can serve as a tool to spatially organize membrane-associated proteins in bottom-up reconstitution studies. For instance, protein condensates and actin networks have been shown to preferentially associate with either L_o_ or L_d_ phases.^8,9^

However, the efficiency of cholesterol incorporation in GUVs is highly dependent on the preparation technique. Using mass spectrometry, a recent study has shown that swelling-based vesicle preparation methods showed a robust and reproducible integration of cholesterol into the bilayer with deviations of less than 5 mol% from the stock lipid mixture. In contrast, the GUVs made by the emulsion-transfer method contained less than 20% of the cholesterol expected from the lipid stocks.^10^ Even poorer cholesterol incorporation (<10%) has been reported for cDICE,^11^ another emulsion-based method. A modified version of the cDICE method (double layer cDICE), where an additional silicone oil layer (free of mineral oil) with the cholesterol dissolved was added to the standard cDICE protocol, was reported to increase the incorporation of cholesterol into GUVs, but still only to about 25–30% of the total amount added to the lipid mixture.^12^ Emulsion-based methods for vesicle fabrication have important advantages over swelling-based methods because they allow for accurate encapsulation of cytosolic contents, and are therefore popular for complex reconstitutions such as efforts to build synthetic cells.^13^ Unfortunately, the poor cholesterol incorporation limits the ability of emulsion-based vesicles to reproduce the biophysical characteristics of native cell membranes.

While mass spectrometry allows the precise quantification of different lipid species in the membrane, it has the drawback of being destructive. Moreover, it provides an ensemble-averaged readout and cannot be used to test for heterogeneities in vesicle populations. An interesting alternative probe of lipid composition is provided by environment-sensitive fluorescent probes that respond to changes in lipid packing and lateral pressure by altering their fluorescence lifetime or spectral profile. Solvatochromic probes, such as Laurdan, Pro12A, di-4-ANEPPDHQ and Nile Red derivatives (NR12S, NR12A and NR4A) have been extensively used to quantify lipid packing within lipid membranes.^14–19^ These probes show a spectral shift in response to the polarity of their local environment. In lipid membranes the polarity represents the hydration level, which is directly related to the membrane order. Saturated lipids pack tightly and leave less space for water molecules at the interface between the hydrophobic and hydrophilic regions of the membrane while unsaturated lipids pack more loosely, creating more disordered membranes that allow more water to enter this region.^20^ A different but complementary environment-sensitive probe is the planarizable push–pull probe Flipper-TR. Contrary to the solvatochromic dyes that respond to polarity, Flipper-TR is a mechanosensitive probe that changes conformation in response to lateral pressure in the bilayer. Its transition from a twisted to a planar conformation is accompanied by an increase in its fluorescence lifetime.^21^ Flipper-TR has been used to investigate lipid packing and phase behaviour in model membranes and as a membrane tension reporter in living cells.^17,22^

In this study, we use a recent modification of the cDICE method called emulsion droplet interface crossing encapsulation (eDICE)^23^ to create GUVs. Compared to cDICE, eDICE is faster, simpler (as it does not require the use of a capillary, which can get clogged, to deliver the droplets) and requires 3 to 4 times lower volume of inner aqueous solution.^23^ After vesicle formation, we used methyl-β-cyclodextrin–cholesterol (MβCD–CL) complexes, a method previously shown to enable cholesterol insertion into membranes,^11^ to deliver cholesterol into the GUVs. To assess the extent of cholesterol incorporation, we employ a combination of *in situ* fluorescence microscopy techniques using environment-sensitive probes that report on lipid packing and membrane order. This approach provides a quantitative and minimally invasive method to quantify the extent of cholesterol integration in the membranes with the final aim to achieve a more accurate representation of biological membranes in vesicle-based model systems.

## 2. Materials and methods

### 2.1. Materials

Mineral oil (M5904), chloroform (372978), biotinylated bovine serum albumin (A8549), poly-vinyl-alcohol (PVA) 145 kDa (8.14894), and methyl-β-cyclodextrin (MβCD) (C4555) were purchased from Sigma. Decane (10497480) and neutravidin (10443985) were bought from Thermo Fisher Scientific. Silicone oil 5cSt (7844) was ordered from Carl Roth. Lipids, specifically cholesterol (ovine) (700000P), 1,2-dimyristoyl-*sn-glycero*-3-phosphocholine (DMPC, 14 : 0 PC) (850345), 1,2-dioleoyl-*sn-glycero*-3-phosphocholine (DOPC, 18 : 1(Δ9-Cis) PC) (850375), 1-stearoyl-2-myristoyl-*sn-glycero*-3-phosphocholine (PC (18 : 0–14 : 0)) (850464), and 1,2-dioleoyl-*sn-glycero*-3-phosphoethanolamine-*N*-(biotinyl) (sodium salt) (Biotin-PE), were purchased from Avanti Polar Lipids and used as supplied. Flipper-TR (SC020) from Spirochrome AG and MemGlow NR12A (MG07) from Cytoskeleton, Inc. were supplied by Tebu Bio.

### 2.2. Gel assisted swelling

To make GUVs using the gel swelling method we followed a protocol described before.^24^ We prepared a 5% (w/v) solution of poly-vinyl alcohol (PVA) in a solution of 200 mM sucrose in milliQ water. We coated a 24 × 24 mm glass coverslip with 100 µL of the PVA solution and removed the excess by tilting the glass on a tissue to minimize gel thickness. The PVA-coated coverslip was heated at 50 °C for 30 minutes in an oven to dry the gel. Then, 10 µL of the lipid mixture (with different compositions, see below) dissolved in chloroform at a total lipid concentration of 1.25 mM were spread on the dried PVA. The organic solvent was evaporated by placing the coverslips under vacuum for 30 minutes. Next, 300 µL of a swelling buffer containing 15 mM Tris pH 7.4 and 140 mM sucrose (175 mOsm kg^−1^) were carefully added on top of the gel. After 1 hour incubation at room temperature, the GUVs were collected and diluted 6-fold in observation buffer (15 mM Tris pH 7.4 and 170 mM glucose, 200 mOsm kg^−1^). The osmolarity of the buffers was measured with a freezing point osmometer (Osmomat 3000, Gonotec, Germany). One set of lipid mixtures used for the gel swollen GUVs was based on DOPC : cholesterol in molar ratios of 100 : 0, 90 : 10, 75 : 25 and 60 : 40. In addition we also prepared samples with binary lipid mixtures of DOPC : DMPC (60 : 40) and DOPC : PC (18 : 0–14 : 0) (60 : 40), and ternary lipid mixtures of DOPC : DMPC : cholesterol (36 : 24 : 40) and DOPC : PC(18 : 0–14 : 0) : cholesterol (36 : 24 : 40). The GUVs were used for confocal imaging within 3 hours after fabrication.

### 2.3. eDICE

To produce GUVs using emulsion droplet interface crossing encapsulation (eDICE) we followed a previously described method.^23^ eDICE is an adaptation of the original cDICE method^25^ where, instead of using a capillary to produce water-in-oil emulsion droplets, we use mechanical agitation to create the droplets. Briefly, the desired lipids were mixed in chloroform and dried under a N_2_ stream. We used three different lipid mixes: DOPC : biotin-PE (99 : 1 mol%), DOPC : PC(18 : 0–14 : 0) : biotin-PE (59 : 40 : 1 mol%) and DOPC : DMPC : biotin-PE (59 : 40 : 1 mol%). The amount of lipids dried (1.75 µmol) yields a final lipid concentration in the lipid-in-oil-mix of 0.25 mM. The vials with the dried lipids were transferred into a glovebox to keep the humidity of the environment below 1%, as this has been reported to improve the quality of the membranes and the vesicle yield.^26^ Once in the glovebox, the lipids were resuspended in 50 µL of chloroform, 400 µL of decane and 6.5 mL of a mix of silicone oil and mineral oil (8 : 2 vol/vol). The vials were sealed and taken out of the glove box, and the lipid-in-oil mixtures were sonicated for 15 minutes on ice in a bath sonicator. The GUVs were prepared using a custom-made spinning table and a 3D-printed chamber described in a previous publication^26^ in a room where the humidity was kept below 40%. We placed the chamber on the spinning table and made it rotate at 2000 rpm. We added 700 µL of an outer aqueous solution (OAS) containing 190 mM glucose (200 mOsm kg^−1^) into the spinning chamber followed by 5 mL of the lipid-in-oil mix. Then, 1 mL of the lipid-in-oil mix was set aside into a 2 ml Eppendorf tube. To create the droplet emulsion we added 25 µL of the inner aqueous solution (IAS) to the tube, with a composition chosen to be compatible with protein encapsulation for future studies (20 mM Tris-HCl pH 7.4, 50 mM KCl, 2 mM MgCl_2_ and 6.5% vol/vol Optiprep, 175 mOsm kg^−1^). We manually created the emulsion by rapidly and vigorously scraping the Eppendorf tube from left to right over an Eppendorf tube holder 10–15 times (alternatively, the emulsion can be made by rapidly pipetting up and down the content of the Eppendorf tube until it becomes foamy). The emulsion was pipetted into the spinning chamber and centrifuged for 3 minutes. Once stopped, we carefully pipetted off the excess of oil from the chamber and added 250 µl of 60 mM Tris-HCl pH 7.4 and 100 mM glucose solution isosmotic with the OAS (200 mOsm kg^−1^) to stabilize the pH of the outer solution. The chambers were kept tilted at an angle of approximately 45° for 10 minutes to allow the GUVs to sediment to the bottom. The GUVs were then collected from the bottom of the spinning chamber with a 200 µL pipette with a cut-off tip to minimize shear forces and transferred to a fresh Eppendorf tube. Finally, the GUVs were diluted by a factor of 4 in observation buffer (15 mM Tris-HCl pH 7.4 and 170 mM glucose). The GUVs were used for confocal imaging experiments within 3 hours after fabrication.

### 2.4. GUV labelling

The fluorescent dyes were dissolved in dimethylsulfoxide (DMSO) following the manufacturer's instructions (Flipper-TR stock at 1 mM and NR12A at 20 µM) and stored in small aliquots at −20 °C. In all the experiments presented here, the GUVs were labelled after being formed by incubating them for 15 minutes with the fluorescent dye of interest. From the dye stock solutions, we added the appropriate volume to the sample of GUVs so the final concentration of the dye in the sample was 1 µM for Flipper-TR (following the manufacturer's recommendation) and 0.1 µM for NR12A (in line with previous reports^17,27^).

### 2.5. Cholesterol delivery to the membrane

Methyl-β-cyclodextrin (MβCD) was preloaded with cholesterol following a previously described protocol.^11^ We first dissolved 2 mg of cholesterol and 55 mg of MβCD in 500 µl of methanol : chloroform (4 : 1 vol/vol) in a 8 mL glass vial and then dried the mixture under nitrogen followed by vacuum for one hour. This results in the formation of crystals at the bottom of the tube. The crystals were resuspended in 2 mL of observation buffer heated at 80 °C by vortexing vigorously until they were completely resuspended, resulting in a suspension of MβCD–cholesterol complexes at a concentration of 2.6 mM cholesterol (1 mg ml^−1^) and 8.3 mM MβCD (11 mg ml^−1^). The suspension was stored at −20 °C and used for no longer than two weeks.

The GUVs were incubated with the MβCD–CL complexes for 15 minutes before imaging. For clarity, we refer to the concentration of cholesterol added to the GUVs, but we emphasize that the cholesterol is always added complexed with MβCD. In our experiments we incubated the GUVs with 10 µM cholesterol (32 µM MβCD), 30 µM cholesterol (96 µM MβCD) and 100 µM cholesterol (320 µM MβCD). Note that we assume that all cholesterol is complexed with MβCD. The results shown across the paper are obtained using different freshly prepared MβCD–cholesterol stocks and showed reproducible results.

### 2.6. Microscopy

All confocal spectral imaging and fluorescence lifetime microscopy measurements were performed on an inverted Leica STELLARIS 8 FALCON laser scanning confocal microscope equipped with a white light laser and a 63× glycerol immersion objective (HC PL APO, WD0.3 mm, NA 1.2). GUVs were observed in Ibidi 18-well µ-slides (Ibidi catalog # 81817). To prevent rupture of the GUVs and reduce drift while imaging, the slides were treated with a solution of biotinylated bovine serum albumin (BSA–biotin, 1 mg mL^−1^ in milliQ water) for 15 minutes, followed by washing with observation buffer and another 10 min incubation with a neutravidin solution (1 mg mL^−1^ in observation buffer). Afterwards, the slide was washed twice with observation buffer to remove unbound neutravidin. The wells in the microscope slide were left filled with 50 µL of observation buffer to prevent drying of the surface. The GUVs (50 µL) were added directly to the observation buffer in the well and allowed to sink down for 5 minutes before imaging.

### 2.7. Confocal spectral imaging and GP analysis of Nile Red 12A

To quantify the generalized polarization (GP) parameter of the NR12A probe within the membrane of the GUVs we performed confocal spectral imaging.^14,28^ The GUVs labelled with NR12A were excited at 540 nm (laser power 15%) and the emission was acquired between 560 and 700 nm with a spectral step of 10 nm per channel using the lambda scan function of the microscope (Fig. S1). The emission was collected using a HyD detector set to counting mode (internal gain 2.5%). The scan speed was set to 700 Hz and the image resolution to 256 × 256 pixels.

The generalized polarization (GP) was computed from the fluorescence spectral images using a custom Python script based on standard libraries, including NumPy, scikit-image, pandas, matplotlib, and tifffile (see Data availability). Prior to GP analysis, the fluorescence spectral images were pre-processed using the StackReg plugin of Fiji to ensure that the GUVs are in the same position in every channel of the image.^29^ The images were loaded as tiff stacks and each stack was converted to a 2D overlay image using maximum intensity projection to create a mask. High-intensity outliers (*e.g.*, saturated pixels) were excluded by applying a brightness threshold. The resulting image was contrast-adjusted based on user-defined percentiles and segmented using automatic thresholding *via* the Otsu algorithm to generate a binary mask. This mask was used to isolate relevant regions for GP analysis, excluding bright artifacts.

Pixel-wise fluorescence intensities were extracted from the selected slices of the stack corresponding to the two wavelengths of interest (570 nm and 640 nm). The intensities at the selected wavelengths were used to calculate the pixel-wise generalised polarization (GP) according to the following equation:^14^1
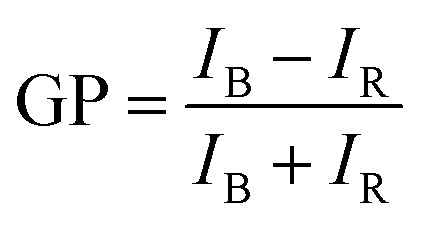
where *I*_B_ is the fluorescence intensity at the shorter wavelength and *I*_R_ is the fluorescence intensity at the longer wavelength. The GP values were mapped back onto the original image geometry to generate GP images. From the pixel-wise GP values we calculated the mean GP of the vesicle in the image.

### 2.8. Fluorescence lifetime microscopy and analysis for Flipper-TR

For FLIM measurements of Flipper-TR, samples were excited at 488 nm using 50% laser power. Emission was collected in the 575–625 nm range using a HyD detector in photon-counting mode (internal gain 10%). The laser power was adjusted to ensure a photon count rate below 0.5 photons per excitation pulse to minimise pile-up artifacts where more than 1 photon per pulse are detected. To ensure the acquisition of enough photons per pixel to obtain reliable decay fits without affecting the photon rate per pulse, we used 5 frame repetitions per image. All acquisitions were performed at a laser repetition rate of 20 MHz.

Fluorescence lifetime analysis was conducted using the Leica Application Suite LAS X FLIM/FCS software (version 4.5.0). Fluorescence decay curves were fitted using a double-exponential reconvolution model, employing the instrument response function (IRF) generated by the FALCON FLIM module within a fitting window of 0.2–45 ns.^17^ Reported lifetime values correspond to the mean intensity-weighted lifetime (*τ*_m int_), calculated by the software according to the following equation:^17^2
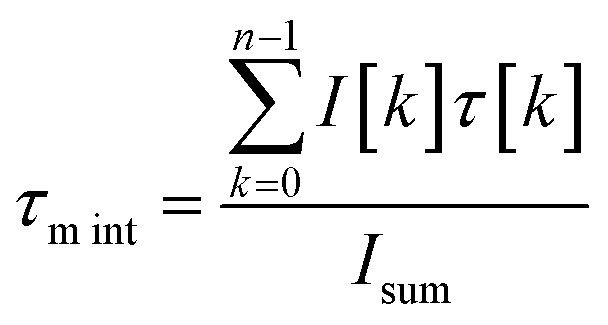
where *n* is set to 2, *I*[*k*] is the intensity of each exponential component, *τ*[*k*] the corresponding lifetime, and *I*_sum_ the total fluorescence intensity.

Phasor analysis was also carried out using the LAS X FLIM/FCS software. A wavelet filter with a threshold of 50 was applied to enhance the distinction of photon clouds in the phasor plot. For quantification, the centre of each photon cloud was manually selected using the circular selection tool with a radius of 20.

### 2.9. Statistics and calibration curves

Spectral and lifetime data for eDICE GUVs with different amounts of MβCD–CL added in the outer medium were based on at least 50 GUVs from 3 different replicates per lipid composition. Prior to statistical comparison, the data were evaluated for normality and homogeneity of variance. As these conditions were not met, we used a non-parametric Kruskal–Wallis ANOVA test to evaluate the statistical significance between the samples incubated with different concentrations of MβCD–CL.

For the calibration analysis, spectral and lifetime measurements were acquired from gel-swollen GUVs prepared with defined cholesterol concentrations. Each condition included at least 30 GUVs from 3 different replicates per lipid composition. The resulting lifetime (Flipper-TR) or GP values (*y*) as a function of MβCD–CL concentration added in the outer medium (*x*) were fitted by a linear regression (*y* = *c* + *mx*) and the fitting parameters *c* and *m* were subsequently used to estimate the effective cholesterol content in the eDICE GUVs following incubation with varying concentrations of MβCD–CL.

All statistical analyses and curve fittings were performed using OriginPro (OriginLab, Northampton, MA, USA).

## 3. Results and discussion

### 3.1. Cholesterol delivery by MβCD into DOPC GUVs made by eDICE can be sensed by NR12A and Flipper-TR probes

We first investigated the incorporation of cholesterol in GUVs made of a single lipid species (DOPC) that forms a homogeneous fluid membrane. To deliver cholesterol into the membrane of GUVs prepared by the eDICE method we incubated the vesicles with different concentrations of MβCD–CL complexes following a previous report.^11^ For clarity we will indicate the concentration of cholesterol in the sample when we added the MβCD–CL complexes. The degree of cholesterol incorporation into the membranes was assessed *in situ* by measuring changes in the lipid packing and lateral pressure through two complementary environment-sensitive fluorescence probes.

**Fig. 1:**
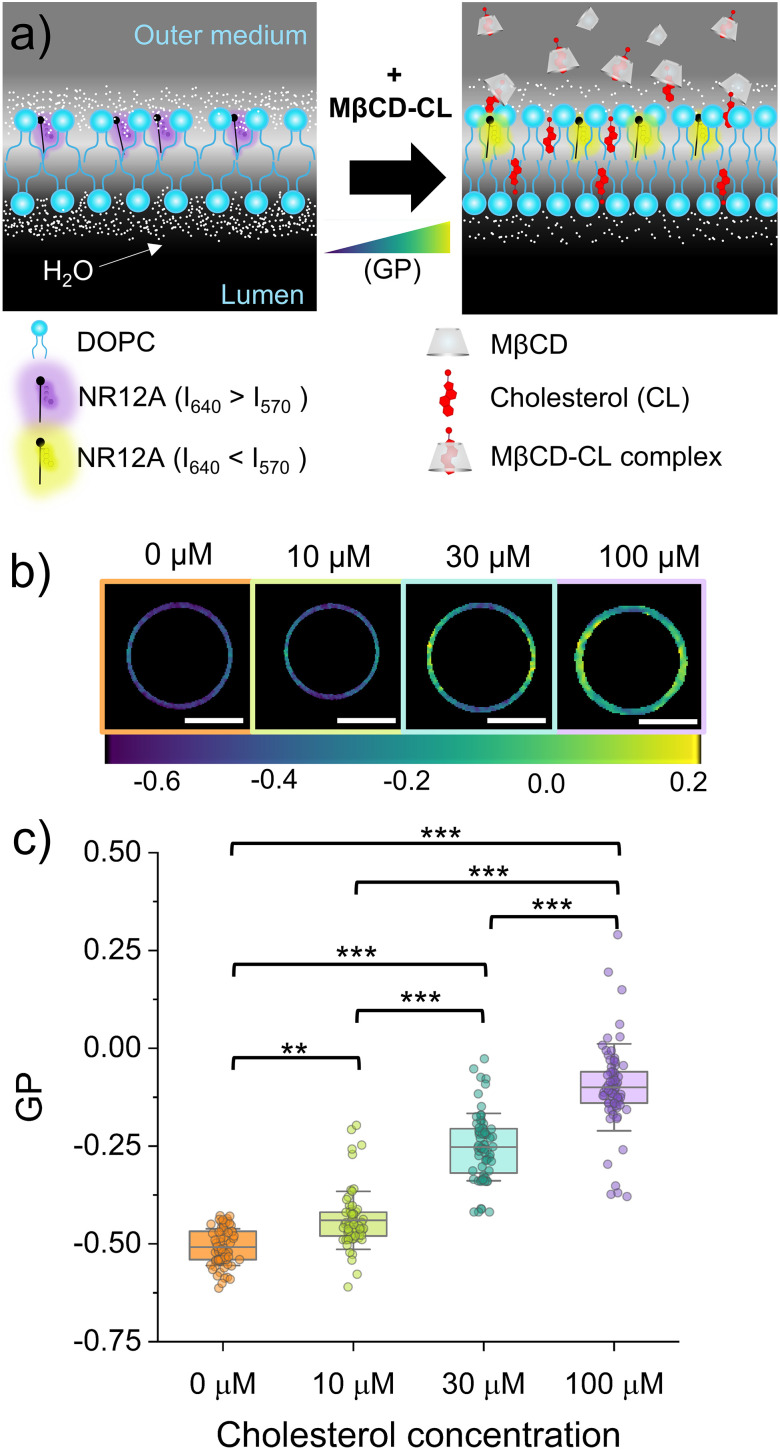
NR12A generalized polarization (GP) as a reporter of cholesterol incorporation in DOPC GUVs. (a) Schematic representation of the change in GP of NR12A in DOPC membranes upon MβCD-driven delivery of cholesterol in the outer medium. (b) Representative color-coded GP images generated from confocal images at the equatorial plane of eDICE GUVs at different cholesterol (MβCD–CL) concentrations. Scale bars indicate 10 µm. (c) GP measured in eDICE GUVs at the different conditions. Box percentile 5–95, line in box is the mean and whiskers the SD. Each data point represents the average GP value of one individual GUV (we measured at least 50 GUVs per condition from at least 3 different replicates). Statistical significance was tested using a non-parametric Kruskal–Wallis test (** *p* < 0.01 and *** *p* < 0.001).

The incorporation of cholesterol in the membrane of the eDICE-produced GUVs upon incubation with MβCD–CL complexes was first evaluated by looking at changes in the generalised polarization (GP) of the polarity sensitive probe NR12A using spectral confocal imaging. The incorporation of cholesterol in the DOPC membrane is expected to increase the lipid packing,^30,31^ leading to a more hydrophobic environment around the NR12A molecules that should result in a spectral shift of the fluorescence emission of the dye towards lower wavelengths^16^ (Fig. 1a). Our spectral imaging measurements using NR12A indeed reveal a clear, concentration-dependent increase in the generalized polarization (GP) values of DOPC GUVs upon the addition of MβCD–CL complexes to the outer medium (Fig. 1b and c). The GP images show different GP values along the membrane of the GUVs (Fig. 1b). These differences are not due to inhomogeneous membrane order but result from an imaging artifact known as photoselection.^28,32^ The excitation of the probe with linearly polarized light leads to a brighter fluorescence emission of NR12A in regions aligned in parallel with the light polarization and dimmer fluorescence in perpendicular regions. These brightness variations translate into artificial GP inhomogeneities. Importantly, the same effect is consistently observed in all GUVs, independent of the sample condition. GP values were averaged along the vesicle contour, which is expected to smooth out orientation-dependent variations while preserving the relative changes between conditions. The averaged GP value of −0.51 ± 0.05 for pure DOPC GUVs reflects a highly disordered membrane environment, typical of unsaturated lipid bilayers.^33^ Upon exposure to 10 µM MβCD–CL, the GUVs show a subtle increase of the mean GP (reaching −0.44 ± 0.08, implying a shift of ΔGP = 0.07 ± 0.09). Further increasing the MβCD–CL concentration results in a further increase of the NR12A GP, reaching mean GP values of −0.25 ± 0.09 (ΔGP = 0.26 ± 0.10) upon incubation with 30 µM cholesterol and −0.10 ± 0.11 (ΔGP = 0.41 ± 0.12) after incubation with 100 µM cholesterol (Fig. 1c and Table 1). This concentration-dependent rise in the GP value clearly suggests that cholesterol has been delivered to the membranes. Compared to the GUVs not exposed to cholesterol, the distribution of the GP values in the samples incubated with MβCD–CL is broader and shows some outliers indicating that the cholesterol incorporation level varies across the GUVs in the sample.

**Table 1:** NR12A GP and Flipper-TR fluorescence lifetime of DOPC GUVs produced by eDICE upon incubation with different concentrations of MβCD–CL

MβCD–CL concentration (µM)	NR12A GP	Flipper-TR lifetime (ns)
0	−0.51 ± 0.05	2.96 ± 0.06
10	−0.44 ± 0.08	3.41 ± 0.05
30	−0.25 ± 0.09	4.05 ± 0.13
100	−0.10 ± 0.11	4.37 ± 0.20

**Fig. 2:**
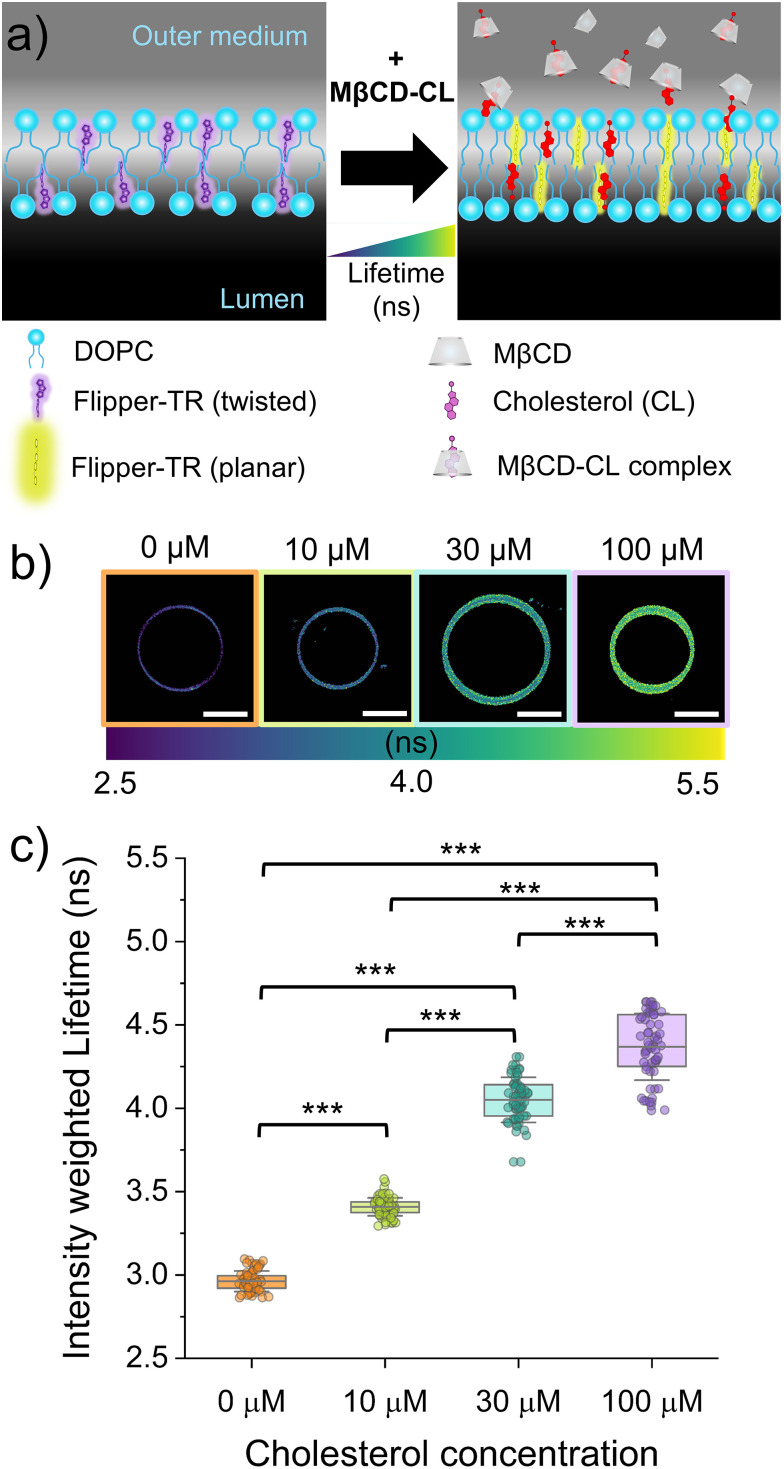
Flipper-TR fluorescence lifetimes as a reporter of cholesterol incorporation in DOPC GUVs. (a) Schematic representation of the conformational change resulting in longer fluorescence lifetimes of Flipper-TR in DOPC membranes upon MβCD-driven delivery of cholesterol in the outer medium. (b) Representative color-coded mean intensity weighted lifetime images at the equatorial plane of eDICE GUVs at different cholesterol (MβCD–CL) concentrations. Scale bars indicate 10 µm. (c) Mean intensity weighted lifetimes of eDICE GUVs as a function of the MβCD–CL concentration in the outer medium. Box percentile 5–95, line in box is the mean and whiskers the SD. Each data point represents the average lifetime value of one individual GUV (we measured at least 50 GUVs per condition from at least 3 different replicates). Statistical significance was tested using a non-parametric Kruskal–Wallis test (*** *p* < 0.001).

To independently verify our observations, we used a complementary approach based on fluorescence lifetime microscopy (FLIM) in combination with the Flipper-TR probe to detect the addition of cholesterol into the membranes. Unlike the NR12A probe that responds to polarity, Flipper-TR responds to changes in membrane packing.^21^ Based on earlier studies of GUVs prepared by swelling, the incorporation of cholesterol in the DOPC membrane is expected to increase the lipid packing^30,31^ and thereby increase the Flipper-TR fluorescence lifetime (Fig. 2a). In all conditions the fluorescence lifetime was homogeneous along the membrane of the GUVs (Fig. 2b). The fluorescence lifetime of Flipper-TR in DOPC GUVs increased progressively with higher concentrations of MβCD–CL, from 2.96 ± 0.06 ns in untreated vesicles to 4.37 ± 0.20 ns after incubation with 100 µM cholesterol (Fig. 2b and c) (Table 1). Additionally, we prepared a control sample of eDICE GUVs with cholesterol incorporated in the lipid solution (DOPC : Cholesterol, 60 : 40 mol%) and did not observe any change in the Flipper-TR fluorescence lifetime (Fig. S2), confirming that the cholesterol in the lipid mixture did not get incorporated in the membrane of eDICE GUVs.

Throughout all tested MβCD–cholesterol concentrations and over the duration of our observations, the GUVs remained intact with no evident membrane rupture or membrane deformations. Delivery (and extraction) of phospholipids *via* MβCD into (and from) the membrane of GUVs has been seen to induce morphological remodelling of the vesicles due to the lipid asymmetry generated in their membrane.^34^ Computational and experimental studies have also linked asymmetric distribution of cholesterol between leaflets to membrane curvature changes.^35,36^ According to those studies, persistent cholesterol asymmetry induced by delivery could perturb the shape of the GUVs, a phenomenon we did not observe, even if the cholesterol was added only to the outer side of the membrane. This is likely due to the known fast flip-flop of cholesterol in fluid lipid bilayers, which allows equilibration between the two leaflets on timescales ranging from milliseconds to minutes.^37–39^

To estimate the cholesterol content in eDICE GUVs after incubation with varying concentrations of MβCD–CL complexes, we used an indirect calibration approach. We prepared control GUVs by gel swelling made of DOPC combined with defined proportions of cholesterol (0%, 10%, 25%, and 40%). These control vesicles provide reference membranes of known composition to relate the fluorescence response of the probes to cholesterol fraction. Swelling methods are known to enable membrane formation from diverse lipid mixtures, including synthetic, charged, and natural lipids, as well as high cholesterol contents.^13^ For instance, swelling assisted by an electric field (electroformation) has previously been shown to give complete incorporation with a deviation from the stock solution lower than 5%.^10^ The range of cholesterol molar ratios used are within physiological levels to ensure the homogeneous distribution of cholesterol and prevent the formation of cholesterol bilayer domains (CBDs), crystals and non-bilayer phases that can appear when the molar ratio of cholesterol approaches its solubility limit known to be around 60 mol%.^40–43^

**Fig. 3:**
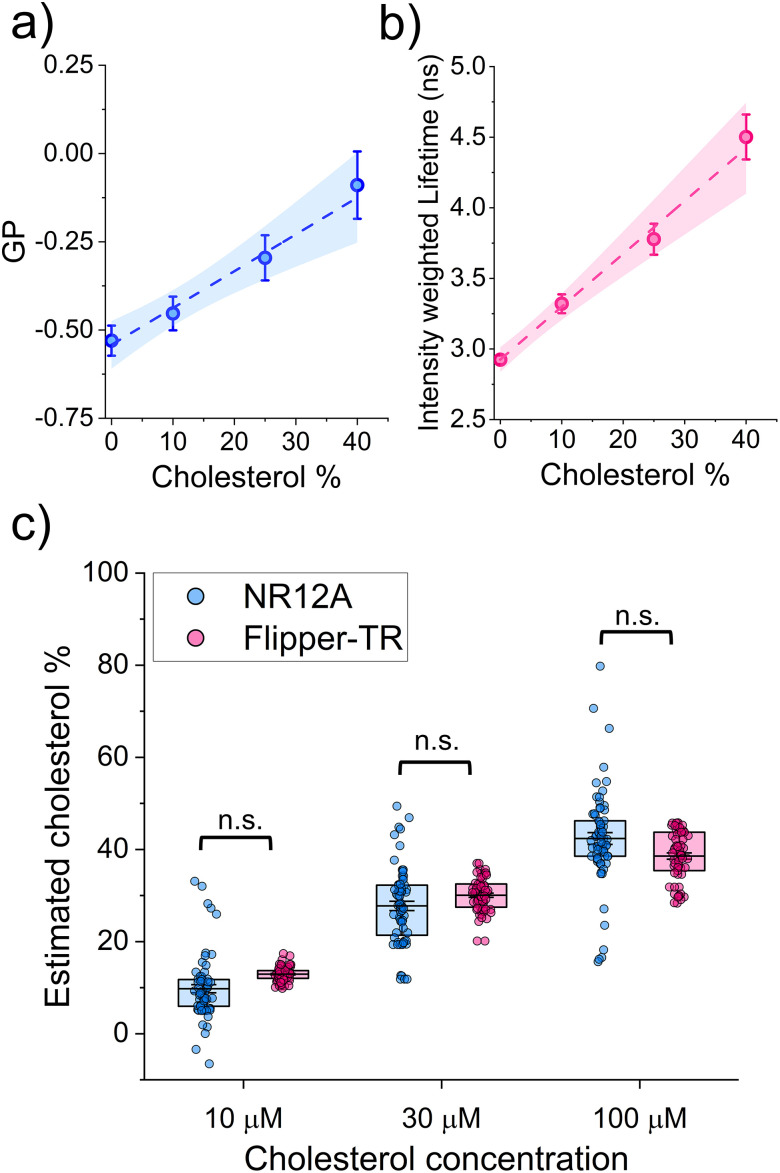
Calibration of fluorescence lifetime and GP measurements of the cholesterol content of GUVs. (a) Mean GP of DOPC GUVs containing different proportions of cholesterol prepared by gel swelling. The dashed line with shaded outline represents the linear regression fit (*y* = *a* + *bx*; *a* = −0.542 ± 0.016 and *b* = 0.010 ± 0.001). (b) Mean intensity weighted lifetimes of DOPC GUVs and different proportions of cholesterol prepared by gel swelling. The dashed line represents the linear regression fit (*y* = *a* + *bx*; *a* = 2.925 ± 0.020 and *b* = 0.037 ± 0.002). In both panels a and b, dots and whiskers represent the mean ± SD (with at least 30 GUVs from 3 different replicates measured per lipid composition) and the shadowed area is the 95% confidence interval of the fit). (c) Cholesterol percentage in the membrane of eDICE GUVs as a function of the MβCD–CL concentration in the outer medium estimated from the calibration curves from gel swollen vesicles, measured by NR12A spectral imaging (based on the data in Fig. 1) and Flipper-TR fluorescence lifetime (based on the data in Fig. 2). Statistical analysis (non-parametric Kruskal–Wallis test) confirms that there is no significant difference (n.s.) in the results obtained from each method. Points represent the cholesterol molar percentage estimated for each single GUV. Box percentile 5–95, line in box is the mean and whiskers the propagated errors.

The corresponding GP values obtained for the gel-swollen samples are presented in Table 2. Similar changes in the GP of NR12A have been reported before in electroformed GUVs made of POPC and increasing proportions of cholesterol.^27^ In that study they observed that, compared to pure POPC vesicles, the presence of 10% of cholesterol in POPC GUVs results in a mild spectral shift of NR12A (ΔGP = 0.06) whereas GUVs containing 50% cholesterol exhibited a substantially increased GP value (ΔGP = 0.54).^27^ To obtain a reference to interpolate the cholesterol levels in the eDICE GUVs based on their measured GP values, we fitted the data for the electroformed GUVs to a linear regression (Fig. 3a). In this way, we calculated that the molar fraction of cholesterol in the GUVs incubated with 10 µM MβCD–CL is 9.78 ± 0.89%, after incubation with 30 µM MβCD–CL it is 27.75 ± 1.06%, and in the vesicles incubated with 100 µM MβCD–CL it is 42.38 ± 1.36% (Fig. 3c and Table 3). Note that the errors reported for the estimated average cholesterol molar percentage are propagated taking into account the errors of the intercept and the slope of linear regression fit as well as the standard deviation of the data points (see SI). In a similar way as with NR12A, we again used GUVs made by gel swelling as a calibration curve for the fluorescence lifetime of Flipper-TR (Fig. 3b and Table 2). From that we estimated the following molar fraction of cholesterol in the GUVs after incubation with the different concentrations of MβCD–CL: 10 µM MβCD–CL = 12.92 ± 0.16%; 30 µM MβCD–CL = 30.07 ± 0.45%; and 100 µM MβCD–CL = 38.57 ± 0.68% (Fig. 3c and Table 3). These results closely match those obtained with NR12A, supporting consistent dose-dependent cholesterol incorporation in the eDICE GUVs controlled by the concentration of MβCD–CL added to the outer medium.

**Table 2:** NR12A GP and Flipper-TR fluorescence lifetime of DOPC GUVs produced by gel swelling with different molar ratios of cholesterol

Lipid composition	NR12A GP	Flipper-TR lifetime (ns)
DOPC	−0.53 ± 0.04	2.92 ± 0.03
DOPC : cholesterol (90 : 10)	−0.45 ± 0.05	3.32 ± 0.07
DOPC : cholesterol (75 : 25)	−0.30 ± 0.07	3.78 ± 0.11
DOPC : cholesterol (60 : 40)	−0.09 ± 0.09	4.50 ± 0.16

**Table 3:** Molar % of cholesterol in eDICE DOPC GUVs exposed to different concentrations of MβCD–CL estimated from NR12A GP and Flipper-TR fluorescence lifetime measurements

MβCD–CL concentration (µM)	From NR12A GP	From Flipper-TR lifetime (ns)
10	9.78 ± 0.89%	12.92 ± 0.16%
30	27.75 ± 1.06%	30.07 ± 0.45%
100	42.38 ± 1.36%	38.57 ± 0.68%

In our experiments, the NR12A GP values and Flipper-TR fluorescence lifetimes measured for eDICE GUVs were very similar to those obtained for gel-swollen GUVs of identical lipid composition. This similarity is somewhat unexpected, as emulsion-based GUV preparation methods, such as eDICE, involve the use of oils and solvents which can remain associated with the membrane after vesicle formation and alter its physical properties.^13,44–46^ The close correspondence between the GP and lifetime values observed here suggests that, under our experimental conditions, any residual oil introduced during eDICE formation is either present at very low levels or does not measurably perturb the local polarity, packing, or lateral pressure sensed by NR12A and Flipper-TR, supporting the use of gel-swollen GUVs as a reliable calibration reference.

### 3.2. Cholesterol delivery by MβCD also works efficiently for GUVs made of binary lipid mixtures

To test whether cholesterol delivery to eDICE GUVs also extends to more complex lipid compositions, we also tested cholesterol incorporation in GUVs containing saturated phosphatidylcholine species by preparing binary mixtures of DOPC with either DMPC or PC(18 : 0–14 : 0) at a 6 : 4 mol/mol ratio. DMPC and PC(18 : 0–14 : 0) differ in the length and symmetry of the acyl chains and consequently their melting temperature. DMPC contains two symmetric myristoyl (14 : 0) chains and has a melting temperature of ∼24 °C, while PC(18 : 0–14 : 0) has one longer stearoyl (18 : 0) and one myristoyl (14 : 0) chain, resulting in an asymmetric structure and a higher melting temperature of ∼30 °C.^47–49^

**Fig. 4:**
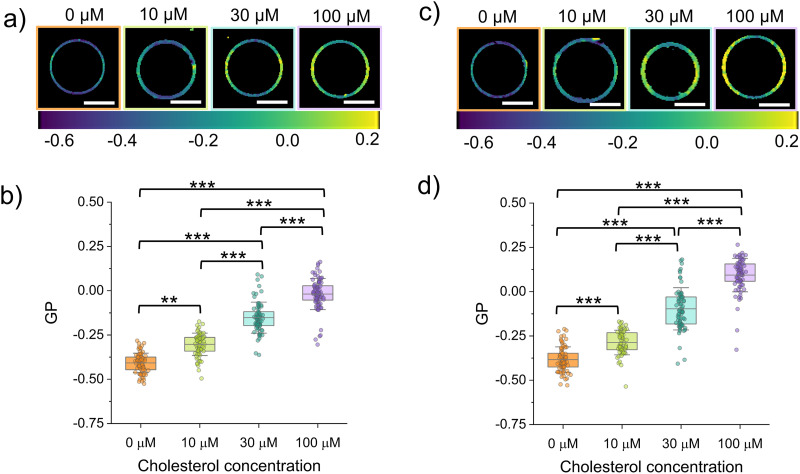
NR12A GP measurements of cholesterol incorporation for eDICE GUVs made of binary lipid mixtures. (a) Representative color-coded GP images of DOPC : PC(18 : 0–14 : 0) (60 : 40 mol%) GUVs at different cholesterol (MβCD–CL) concentrations in the outer medium. (b) GP values of DOPC : PC(18 : 0–14 : 0) GUVs as a function of MβCD–CL concentration. (c) Representative color-coded GP images of DOPC : DMPC (6 : 4 mol%) GUVs at different cholesterol (MβCD–CL) concentrations in the outer medium. (d) GP values of DOPC:DMPC GUVs as a function of MβCD–CL concentration. Scale bars in panels a and c indicate 10 µm. In panels b and d, box represents percentile 5–95, line in box is the mean and whiskers the SD. Each data point represent the average GP value of one individual GUV (we measured at least 50 GUVs per condition from at least 3 different replicates). Statistical significance was tested using a non-parametric Kruskal–Wallis test (** *p* < 0.01 and *** *p* < 0.001).

**Fig. 5:**
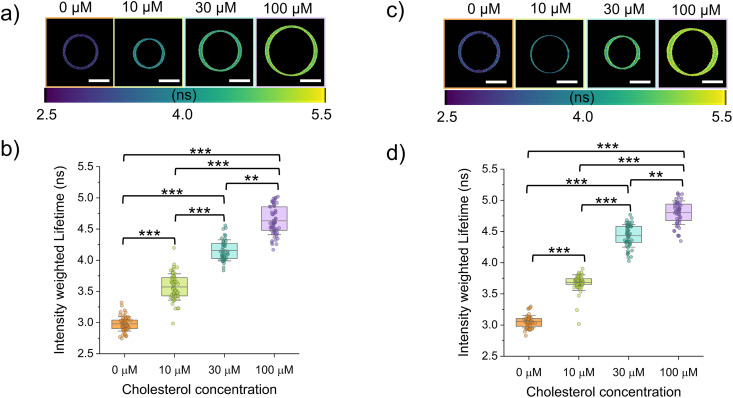
Flipper-TR fluorescence lifetimes as a reporter of cholesterol incorporation in eDICE GUVs made of binary lipid mixtures. (a) Representative color-coded mean intensity weighted lifetime images of DOPC : PC(18 : 0–14 : 0) (60 : 40 mol%) GUVs at different cholesterol (MβCD–CL) concentrations in the outer medium. (b) Mean intensity weighted lifetimes of DOPC : PC(18 : 0–14 : 0) GUVs as a function of MβCD–CL concentration. (c) Representative color-coded mean intensity weighted lifetime images of DOPC : DMPC (6 : 4 mol%) GUVs at different cholesterol (MβCD–CL) concentrations in the outer medium. (d) Mean intensity weighted lifetimes of DOPC:DMPC GUVs as a function of MβCD–CL concentration. Scale bars in panels a and c indicate 10 µm. In panels b and d, box represents percentile 5–95, line in box is the mean and whiskers the SD. Each data point represents the average lifetime value for one individual GUV (we measured at least 50 GUVs per condition from at least 3 different replicates). Statistical significance was tested using a non-parametric Kruskal–Wallis test (** *p* < 0.01 and *** *p* < 0.001).

Prior to cholesterol addition, the NR12A GP values were −0.38 ± 0.07 for DOPC : DMPC and −0.41 ± 0.05 for DOPC : PC(18 : 0–14 : 0) (Fig. 4a–d), both notably higher than those observed in pure DOPC membranes (−0.51 ± 0.05, Fig. 1c). This is consistent with previous observations using NR12A that have shown that acyl chain saturation increases lipid packing.^27^ In contrast, Flipper-TR-labelled GUVs composed of the same binary mixtures exhibited average fluorescence lifetimes (3.05 ± 0.10 ns for DOPC : DMPC and 2.98 ± 0.11 ns for DOPC : PC(18 : 0–14 : 0)) comparable to that of pure DOPC (Fig. 5a–d). Consistent with these findings, similar NR12A GP values (DOPC : DMPC = −0.37 ± 0.06 and DOPC : PC(18 : 0–14 : 0) = −0.39 ± 0.07) and Flipper-TR fluorescence lifetimes (DOPC : DMPC = 3.07 ± 0.08 ns and DOPC : PC(18 : 0–14 : 0) = 3.01 ± 0.06 ns) were observed in gel-swollen GUVs of equivalent lipid composition (Fig. S1). This discrepancy likely stems from the differing sensing mechanisms of the two probes: while NR12A detects changes in local polarity linked to packing,^16,27^ Flipper-TR responds to lateral pressure changes through a conformational twist.^21,22^ These observations imply that, although saturated lipids promote tighter packing, this effect may not generate sufficient lateral stress to trigger the mechanical response required for a change in Flipper-TR lifetime. Notably, while previous studies have reported solid–liquid domain coexistence in vesicles made of equimolar ratios of DOPC : DMPC and DOPC : PC(18 : 0–14 : 0) with no or low (5%) cholesterol,^50,51^ in our experiments no signs of lateral heterogeneity or phase separation were observed in either system (eDICE and gel-swollen GUVs), probably due to the slightly higher ratio of DOPC used in our GUVs. The distinction between NR12A and Flipper-TR behaviour is supported by previous studies comparing environment sensitive probes. Amaro *et al.*^18^ showed that Laurdan is an accurate and sensitive indicator of lipid order while di-4-ANEPPDHQ is sensitive to the membrane potential and its GP does not correlate with lipid packing but is specifically influenced by the presence of cholesterol. Other previous studies have reported that while both NR12A and Flipper-TR detect cholesterol content, only NR12A exhibits strong sensitivity to lipid saturation whereas Flipper-TR is relatively insensitive to acyl chain ordering.^17,27^

Similarly to what we observed in DOPC GUVs, the exposure to MβCD–CL complexes did not result in any noticeable disruption of the vesicles made from binary lipid mixtures. Upon incubation with MβCD–CL complexes, both the DOPC : PC(18 : 0–14 : 0) and the DOPC : DMPC GUVs show a concentration dependent increase of the NR12A GP (Fig. 4) (Table 4). Upon addition of cholesterol at increasing concentrations to DOPC:DMPC GUVs, the average GP values of NR12A progressively increased from −0.38 ± 0.07 to −0.29 ± 0.07, −0.10 ± 0.12, and 0.09 ± 0.09 (Fig. 4c, d and Table 4). Notably, at the highest cholesterol concentration tested (100 µM), the DOPC : DMPC mixture displayed a higher GP value (NR12A GP = 0.09 ± 0.09) than DOPC : PC(18 : 0–14 : 0) (NR12A GP = −0.02 ± 0.09), despite DMPC having a lower melting temperature. Flipper-TR lifetime measurements followed a similar trend, with the fluorescence lifetime increase upon incubation with increasing cholesterol concentrations being higher for the DOPC:DMPC GUVs (Fig. 5a–d and Table 5). The GP and fluorescence lifetime values obtained after exposure to 100 µM cholesterol are very similar to those observed in gel swollen GUVs made of DOPC : DMPC : cholesterol (36 : 24 : 40) (NR12A GP = 0.07 ± 0.15; Flipper-TR lifetime = 4.78 ± 0.21 ns) and DOPC : PC(18 : 0–14 : 0) : cholesterol (36 : 24 : 40) (NR12A GP = 0.00 ± 0.07; Flipper-TR lifetime = 4.67 ± 0.20 ns) (Fig. S2). These results suggest that the mismatch in chain length in PC(18 : 0–14 : 0) could reduce the efficiency of cholesterol-induced lipid packing. This is consistent with previous findings that have shown that sterols align more efficiently with symmetric saturated lipids whereas asymmetry in acyl chains disrupts this interaction, leading to a diminished effect on membrane order.^52^

**Table 4:** NR12A GP of eDICE GUVs made of binary lipid mixtures upon incubation with different concentrations of MβCD–CL

MβCD–CL concentration (µM)	DOPC : DMPC	DOPC : PC (18 : 0–14 : 0)
0	−0.38 ± 0.07	−0.41 ± 0.05
10	−0.29 ± 0.07	−0.30 ± 0.06
30	−0.10 ± 0.12	−0.15 ± 0.09
100	0.09 ± 0.09	−0.02 ± 0.09

**Table 5:** Flipper-TR fluorescence lifetime (ns) of eDICE GUVs made of binary lipid mixtures upon incubation with different concentrations of MβCD–CL

MβCD–CL concentration (µM)	DOPC : DMPC	DOPC : PC (18 : 0–14 : 0)
0	3.05 ± 0.10	2.98 ± 0.11
10	3.68 ± 0.12	3.57 ± 0.21
30	4.43 ± 0.18	4.15 ± 0.17
100	4.80 ± 0.19	4.63 ± 0.22

**Fig. 6:**
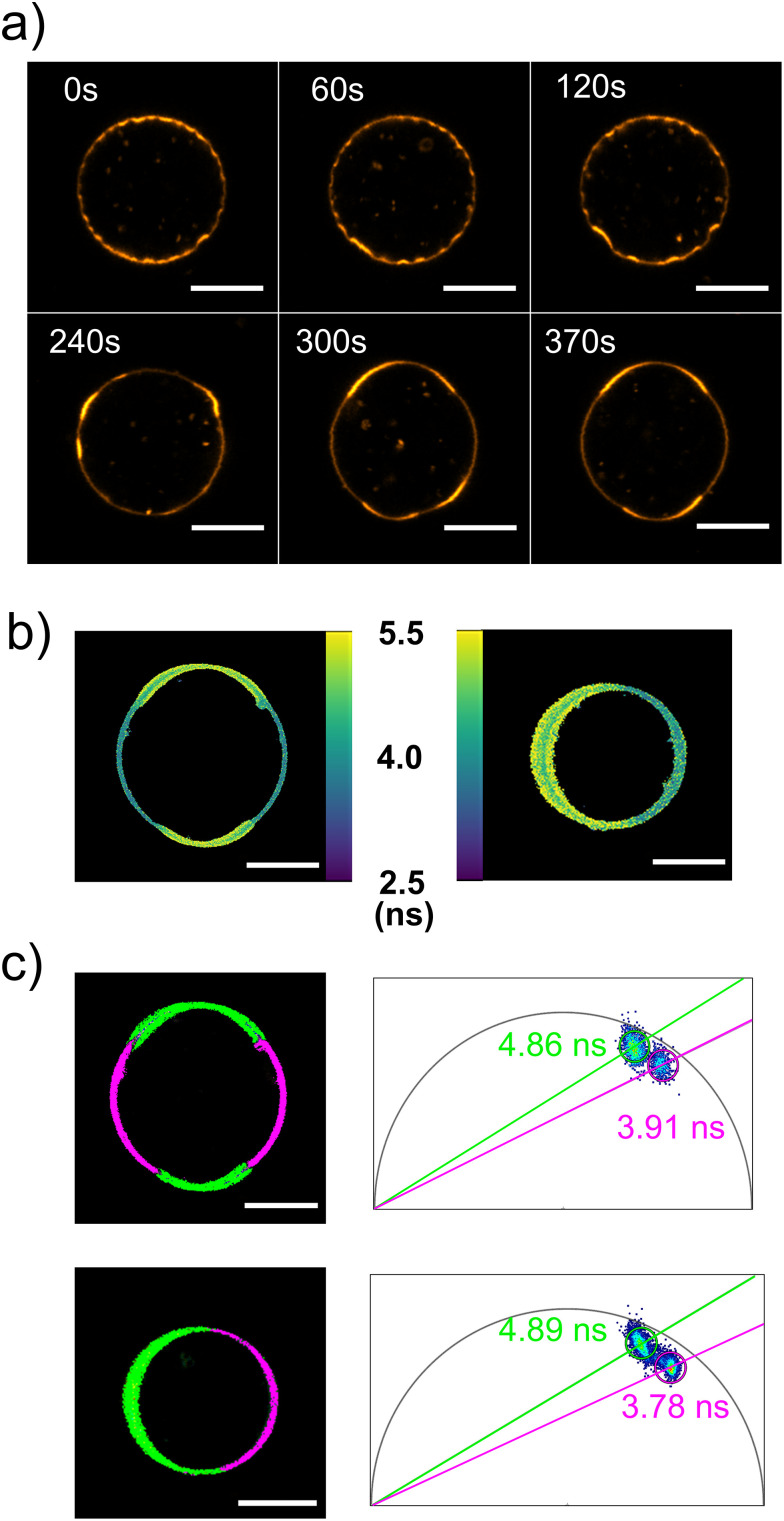
Examples of DOPC : DMPC (6 : 4 mol%) GUVs exhibiting liquid–liquid phase separation upon cholesterol incorporation. (a) Confocal microscopy timelapse images of the evolution of membrane domains of a GUV labelled with Flipper-TR at different time points in the presence of 100 µM cholesterol (*i.e.*, MβCD–CL) in the outer medium. The *t* = 0 indicates the start of the imaging, approximately 15 minutes after the addition of MβCD–CL to the outer medium. Images show the fluorescence intensity signal of Flipper-TR at an emission wavelength between 575 and 625 nm. (b) Color-coded mean intensity weighted lifetime images of two different phase separated GUVs labelled with Flipper-TR recorded within 25 and 45 minutes after addition of MβCD–CL. (c) Phasor analysis of the phase separated GUVs shown in panel b. The images (left) show the regions of the membrane separated by their lifetime according to the clouds on the phasor plots on the right. The lifetime values corresponding to the L_d_ (magenta) and L_o_ (green) domains are indicated in the phasor plots. Scale bars in panels a–c, denote 10 µm.

Interestingly, in our Flipper-TR experiments, we detected a small number of DOPC:DMPC GUVs exhibiting liquid–liquid phase separation after incubation with 30 µM or 100 µM cholesterol. Phase separation was observed in 5 of 115 GUVs analysed, with events observed in 4 of 6 independent experimental replicates. It should be noted that numerous additional vesicles in the samples appeared homogeneous but were not imaged or quantified, thus, these numbers represent only the imaged subset and do not constitute a population-wide frequency estimate. These GUVs initially showed small bright domains with negative curvature that fused into larger and more energetically favourable domains over time (see Fig. 6a and Video S1). The final domains were clearly distinguished by their fluorescence lifetime (Fig. 6b and c). Phasor analysis of the fluorescence lifetime showed that the phase separated GUVs present L_o_ domains with fluorescence lifetimes between 4.83 and 4.91 ns and less packed L_d_ domains with fluorescence lifetimes between 3.78 and 3.99 ns. Although these observations were rare, they suggest that cholesterol enrichment can induce phase separation in this membrane mixture. Adding cholesterol to the outer medium could be interesting for studying the dynamics of membrane organization in response to cholesterol or for triggering recruitment of lipid domain-specific proteins in synthetic cells.^8,53,54^

A recent study has shown that ternary mixtures of DOPC : DMPC : cholesterol at a molar ratio of 40 : 40 : 20 exhibit a miscibility transition temperature of around 18.5 °C.^50^ Our experiments were performed above that temperature (∼21 °C), which is consistent with the largely homogeneous appearance of most GUVs in our samples. However, other previous work reported a higher prevalence of macroscopic phase separation in DOPC : DMPC : cholesterol membranes, using equimolar mixtures of DOPC : DMPC (1 : 1) across a range of cholesterol ratios (5–50%), in the range of temperatures in which our experiments were performed.^51^ In these studies, they observed that ternary membranes that exhibit L_o_/L_d_ coexistence at intermediate sterol fractions may transition into a single liquid-ordered phase when the cholesterol content exceeds 40 mol%.^50,51^ However, in our system we did not observe any evidence of liquid–liquid phase separation at low cholesterol concentrations, nor did we detect gel–liquid phase coexistence prior to cholesterol addition. Therefore, the homogeneous appearance of our GUVs cannot be attributed to cholesterol-induced suppression of phase separation. Compared to those systems with equimolar ratios of DOPC : DMPC, our GUVs contain a lower fraction of DMPC (6 : 4 DOPC : DMPC), which is expected to favour mixing and shift the miscibility transition to lower temperatures. This compositional difference provides a plausible explanation for why phase separation is less frequently observed in our experiments, despite cholesterol concentrations comparable to those reported to induce domain formation in more DMPC-rich membranes. The low frequency of phase separation events in our samples may therefore reflect occasional deviations in lipid composition at the single-vesicle level, for instance GUVs locally enriched in DMPC relative to the expected average composition, which would shift the miscibility transition to higher temperatures and allow macroscopic phase separation under our experimental conditions. Finally, nanoscopic lateral heterogeneities have been reported in GUVs made of both DOPC : DMPC : cholesterol and DOPC : PC(18 : 0–14 : 0) : cholesterol above the microscopic transition temperature.^50^ While the optical resolution of our fluorescence lifetime imaging experiments does not allow us to directly detect such nanoscale domains, we cannot exclude the presence of sub-diffraction heterogeneities in our GUVs, even when membranes appear macroscopically uniform.

### 3.3. Discussion

Cholesterol plays a central role in modulating the biophysical properties of cellular membranes. It is therefore essential to incorporate physiologically relevant cholesterol levels in model membrane systems for studying membrane structure and function. Incorporating cholesterol into GUVs made by swelling is straightforward but swelling-based methods do not allow for accurate encapsulation of cytosolic contents.^6,13^ By contrast, incorporating cholesterol into GUVs made by emulsion-based methods, which are popular for bottom-up synthetic cell reconstitution,^6,13^ is challenging because cholesterol remains dissolved in the oil phase due to its high hydrophobicity.^10^

The use of MβCD as a cholesterol carrier has been well established for modulating membrane cholesterol content, both in live cells^55,56^ and in model membrane systems.^7,11^ MβCD can act as both a donor and an acceptor, depending on its loading state. Our study builds on this principle by demonstrating that MβCD–CL complexes can effectively deliver cholesterol to GUVs produced using the eDICE method with different initial lipid compositions. We demonstrated that cholesterol delivery can be reliably detected using two complementary environment-sensitive fluorescent probes: NR12A and Flipper-TR. Both probes revealed a clear, concentration-dependent incorporation of cholesterol into eDICE GUV membranes, as evidenced by increasing NR12A generalized polarization (GP) values and Flipper-TR fluorescence lifetimes. The GP and fluorescence lifetime values obtained are not a direct measurement of the cholesterol content in the membrane but can be used to infer it using a calibration curve based on GUVs with known cholesterol molar ratio prepared by gel-swelling. We used this method in DOPC GUVs, and demonstrated the tunability of the final cholesterol content of eDICE GUVs over the physiologically relevant range of 10 to 40 mol% by variations in the concentration of MβCD–CL in the outer medium. While the GUVs in our samples did not show clear signs of membrane perturbation upon exposure to MβCD–CL complexes throughout the duration of our experiments, the presence of free uncomplexed MβCD in the external environment could in principle continue to extract lipids (both cholesterol and phospholipids) from the membrane after initial delivery,^55^ therefore developing methods to remove or neutralize free MβCD from the medium would be desirable to prevent potential lipid depletion from the membranes.

We observed subtle differences between the probes in GUVs composed of binary lipid mixtures with saturated lipids (DMPC and PC(18 : 0–14 : 0)). Upon cholesterol enrichment, both probes detected increased membrane order, with stronger responses in the DOPC : DMPC mixture compared to DOPC : PC(18 : 0–14 : 0), likely due to better cholesterol accommodation in bilayers with symmetric saturated chains which translates into a stronger impact on the membrane order.^52^ While we did not perform the full calibration *versus* gel-swollen GUVs to precisely estimate the cholesterol content of these GUVs, the NR12A GP values and Flipper-TR fluorescence lifetimes of the eDICE GUVs after incubation with the highest concentration of MβCD–CL explored were comparable to the ones obtained in gel-swollen GUVs made of DOPC 36 mol%, DMPC or PC(18 : 0–14 : 0) 24 mol% and cholesterol 40 mol%, suggesting that the presence of these saturated lipid species does not affect the incorporation of cholesterol in the membrane.

The cholesterol estimation approach presented here has being tested only in GUVs with simple lipid compositions containing only PC species that remain mostly homogeneously distributed along the membrane and cholesterol levels between 0 and 40 mol%. At higher cholesterol contents, deviations from linearity may arise, as cholesterol is known to approach its solubility limit in PC bilayers at molar fractions above 60 mol%, which can lead to increasingly inhomogeneous lateral distribution rather than ideal mixing.^40–43^ In addition, we observed isolated phase separation events in DOPC:DMPC GUVs at higher cholesterol concentrations, which confirms that cholesterol can promote lateral heterogeneity and domain formation under specific compositional conditions. From studies on vesicles generated by swelling methods, it is well known that ternary lipid mixtures combining an unsaturated low-melting lipid (such as DOPC), a saturated high-melting lipid (such as DPPC or sphingomyelins), and cholesterol frequently display coexisting liquid-ordered (L_o_) and liquid-disordered (L_d_) phases depending on the molar ratio of the lipids and the temperature.^7,57,58^ In such phase separated membranes, cholesterol has more affinity for high *T*_m_ lipids and sphingomyelins so it partitions preferentially into these ordered domains.^58,59^ Within this context, our observations should be viewed as a quantitative GUV-based implementation of these well-established principles in an emulsion-derived vesicle platform rather than as a fundamentally new mechanistic insight into sterol–lipid interactions. Importantly, while the quantitative calibration established here provides a robust baseline for fluid, single-phase membranes, the presence or emergence of membrane domains may require adaptations for the cholesterol estimation, since it will no longer be equally distributed along the membrane. Such analyses could include domain-resolved analysis and more complex fitting models.

Beyond methodological considerations, the ability to controllably introduce cholesterol into emulsion-based GUVs has important implications for synthetic biology and membrane reconstitution studies. Cholesterol is not only a structural modulator of membrane order but also a key regulator of protein–membrane interactions.^60–62^ For instance, recent work has shown that cholesterol enrichment facilitates the recruitment of the ESCRT-0 component HRS and promotes the formation of HRS–clathrin microdomains.^63^ Cholesterol-enriched membranes can therefore be essential for modelling endocytic processes in synthetic cells. Cholesterol also modulates the localization and activity of many membrane proteins through direct recognition motifs, such as CRAC/CARC sequences, and lipid-dependent partitioning effects.^60–62,64–66^ In laterally heterogeneous membranes, it contributes to the organization of raft-like domains that can concentrate palmitoylated proteins and glycolipid-associated components such as GM1, thereby influencing signalling and transport processes.^67^ Moreover, reconstructions of the membrane composition of minimal mycoplasma-derived cells demonstrate how lipid composition critically determines membrane functionality and viability.^68,69^ Within this context, controlled sterol incorporation may enable to reproduce organism-specific membrane compositions, which can be crucial for reconstituting essential cellular processes such as the bacterial division of mycoplasma.^70^ The strategy presented here provides a practical route to incorporate physiologically relevant cholesterol levels into emulsion-based GUV systems, thereby extending their applicability to studies of biological processes where the presence of sterol in the membrane is essential.

## 4. Conclusion

In our study we provide a unified strategy to both manipulate and monitor membrane cholesterol levels into eDICE GUVs *in situ*, by coupling post-formation cholesterol delivery *via* MβCD with non-invasive fluorescence microscopy using the environment-sensitive probes NR12A and Flipper-TR. This combined approach not only confirms the successful enrichment of vesicles with cholesterol but also establishes a broadly applicable method to study sterol-dependent membrane organization under controlled conditions. This method enables emulsion-based GUVs to serve as realistic, cholesterol-rich platforms for reconstitution of lipid–protein interactions and the engineering of synthetic cells.

Future studies could further investigate the emergence of phase separation by exploring a broader range of lipid mixtures, including those with higher melting temperature components such as DPPC or sphingomyelins. In our experience, however, incorporation of such high-melting lipids into membranes using the current eDICE protocol is inefficient, so methodological adaptations, such as vesicle production at elevated temperature or adjustment of oil and solvent composition, may be required to produce such vesicles. Additionally, temperature-controlled experiments could provide critical insight into the thermodynamic conditions that favour domain formation, particularly since all current measurements were performed at room temperature. Importantly, phase-separated GUVs offer an excellent platform for reconstitution studies, where the presence of coexisting membrane domains can be exploited to control and study the spatial organization of membrane-associated proteins and cytoskeletal networks. Such systems could help unravel how lipid heterogeneity contributes to protein localization, membrane mechanics, and cellular signalling processes.

## Author contributions

MAP and GHK devised the project, conceived the main conceptual ideas and discussed the results. MAP designed and performed the experiments and data analysis and wrote the first manuscript draft. GHK contributed to the interpretation of the results, provided critical feedback and helped shape the research, analysis and manuscript.

## Conflicts of interest

There are no conflicts to declare.

## Supplementary Material

SM-022-D5SM01124H-s001

SM-022-D5SM01124H-s002

## Data Availability

Supplementary information (SI): providing an explanation of the error propagation calculations for the reported cholesterol contents, two tables providing data for NR12A GP and Flipper-TR replicate measurements, three figures showing unprocessed fluorescence microscopy images of GUVs labelled with NR12A, lifetime measurements for eDICE vesicles made using a lipid mixture containing cholesterol, and calibration NR12A GP and Flipper-TR measurements for gel-swollen GUVs, and one video showing time-lapse data of a phase-separating GUV upon cholesterol delivery. See DOI: https://doi.org/10.1039/d5sm01124h. Data for this article, including Flipper-TR FLIM measurements and NR12A GP values are available at 4TU. Research data at https://doi.org/10.4121/5a3473d9-56e7-4fe7-ba9b-cd81462ba47f. The python script for the analysis of NR12A GP values of the GUVs is available on https://gitlab.tudelft.nl/marribasperez/gp-analysis-spectral-imaging.git.
