# Supplementary material for: Quantification of cholesterol incorporation in giant unilamellar vesicles produced by a modified cDICE method
Source: Soft Matter. 2026 Mar 5;22(12):2408–21. doi: 10.1039/d5sm01124h (PMC12970526; doi:10.1039/d5sm01124h)
Supplement: SM-022-D5SM01124H-s001 [file SM-022-D5SM01124H-s001.pdf]

Supplementary Information (SI) for Soft Matter.
This journal is © The Royal Society of Chemistry 2026

Supporting Information for:

Quantification of Cholesterol Incorporation in Giant Unilamellar Vesicles Produced by a Modified cDICE Method

Marcos Arribas Perez and Gijsje H. Koenderink

Department of Bionanoscience, Kavli Institute of Nanoscience, Delft University of Technology, 2629 HZ Delft, The Netherlands

Error propagation

1. NR12A GP shift

The NR12A GP shift (*ΔGP*) was calculated as:

$$\Delta \mathrm{GP} = \mathrm{GP}_0 - \mathrm{GP}_x$$

Where GP_0 is the mean NR12A GP of the GUVs before incubation with MβCD-CL and GP_x is the mean NR12A GP of the GUVs upon incubation with a concentration x of MβCD-CL. The error associated to ΔGP (*δΔGP*) is calculated from the standard deviation of GP_0 (δGP_0) and GP_x (δGP_0) using the following equation:

$$\delta \Delta \mathrm{GP} = \sqrt{[(\delta \mathrm{GP}_0)^2 + (\delta \mathrm{GP}_x)^2]}$$

2. Estimation of cholesterol mol% in the membrane of eDICE GUVs

The cholesterol mol% (x) in the membrane of eDICE GUVs was interpolated from a linear calibration obtained from standards of known concentration:

$$y = a + mx$$

where *y* is the measured value (GP or fluorescence lifetime), *m* is the slope of the linear fit and *a* is the intercept. Therefore, the cholesterol mol% (*x*) for every individual GUV measured can be calculated as:

$$x = \frac{y - a}{m}$$

The error of the cholesterol mol% calculated for every individual GUV measured (*δx)* is given by the following equation:

$$\delta x = \sqrt{\frac{\delta a^2}{m^4}} + \frac{(y - a)^2 \delta m^2}{m^4}$$

where *δa* is the error of the intercept and *δm* the error of the slope.

Once we calculated the cholesterol mol% in every GUV measured, we calculated the average cholesterol mol% in the sample (C) as:

$$C = \frac{\sum x}{N}$$

where N is the number of individual GUVs in the sample.

The error of *C (δc)* is obtained by we combined the contribution of the standard deviation (*s*) with the propagation of the errors calculated for the individual GUVs (*δx)* using the following equation:

$$\delta c = \sqrt{\frac{s^2}{N} + \frac{\sum \delta x^2}{N^2}}$$

Table S1. NR12A GP values (mean±sd) averaged per replicate of the experiments for cholesterol delivery into eDICE GUVs of different initial lipid mixtures.

Initial lipid mixture	MβCD-CL concentration (μM)	Replicate	NR12A GP
DOPC	0	1	-0.56 ± 0.04
		2	-0.48 ± 0.04
		3	-0.50 ± 0.03
		4	-0.51 ± 0.06
	10	1	-0.43 ± 0.07
		2	-0.43 ± 0.09
		3	-0.46 ± 0.04
	30	1	-0.26 ± 0.08
		2	-0.25 ± 0.10
		3	-0.25 ± 0.08
	100	1	-0.07 ± 0.08
		2	-0.11 ± 0.14
		3	-0.11 ± 0.08
DOPC:DMPC (6:4)	0	1	-0.40 ± 0.03
		2	-0.40 ± 0.07
		3	-0.38 ± 0.09
		4	-0.35 ± 0.05
	10	1	-0.30 ± 0.04
		2	-0.26 ± 0.06
		3	-0.30 ± 0.09
	30	1	-0.09 ± 0.12
		2	-0.11 ± 0.10
		3	-0.09 ± 0.12
	100	1	0.08 ± 0.09
		2	0.12 ± 0.06
		3	0.08 ± 0.11
DOPC: PC(18:0-14:0) (6:4)	0	1	-0.41 ± 0.05
		2	-0.39 ± 0.03
		3	-0.41 ± 0.06
		4	-0.39 ± 0.06
	10	1	-0.32 ± 0.08
		2	-0.29 ± 0.06
		3	-0.31 ± 0.06
		4	-0.30 ± 0.05
	30	1	-0.17 ± 0.06
		2	-0.15 ± 0.10
		3	-0.15 ± 0.09
		4	-0.16 ± 0.07
	100	1	-0.01 ± 0.08
		2	-0.01 ± 0.04
		3	-0.04 ± 0.10
		4	-0.01 ± 0.09

Table S2. Flipper-TR fluorescence lifetime (mean±sd) averaged per replicate of the experiments for cholesterol delivery into eDICE GUVs of different initial lipid mixtures.			
**Initial lipid mixture**	**MβCD-CL concentration (μM)**	**Replicate**	**Flipper-TR lifetime (ns)**
DOPC	0	1	2.96 ± 0.05
		2	2.98 ± 0.07
		3	2.95 ± 0.06
		4	2.97 ± 0.07
	10	1	3.42 ± 0.06
		2	3.40 ± 0.05
		3	3.41 ± 0.04
	30	1	4.03 ± 0.13
		2	4.07 ± 0.14
		3	4.06 ± 0.13
	100	1	4.38 ± 0.24
		2	4.35 ± 0.21
		3	4.36 ± 0.06
DOPC:DMPC (6:4)	0	1	3.09 ± 0.14
		2	3.07 ± 0.03
		3	3.00 ± 0.07
	10	1	3.65 ± 0.07
		2	3.75 ± 0.07
		3	3.64 ± 0.19
	30	1	4.51 ± 0.29
		2	4.36 ± 0.12
		3	4.43 ± 0.23
	100	1	4.89 ± 0.12
		2	4.73 ± 0.12
		3	4.80 ± 0.24
DOPC: PC(18:0-14:0) (6:4)	0	1	2.96 ± 0.17
		2	2.96 ± 0.03
		3	3.01 ± 0.08
	10	1	3.63 ± 0.22
		2	3.52 ± 0.24
		3	3.57 ± 0.14
	30	1	4.17 ± 0.23
		2	4.04 ± 0.05
		3	4.23 ± 0.09
	100	1	4.68 ± 0.24
		2	4.74 ± 0.16
		3	4.46 ± 0.17

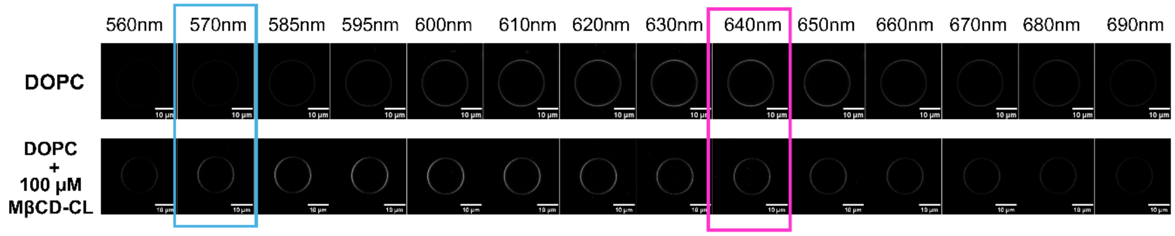

Figure S1. Fluorescence microscopy images of GUVs labelled NR12A before processing, separated by channels. Images were collected between 560 and 700 nm with a spectral step of 10 nm per channel using the lambda scan function of the microscope. We indicate the initial wavelength of each channel on top of the micrographs. The blue and magenta rectangles highlight the channels used in the data analysis to calculate the GP values. The scale bars are 10 μm.

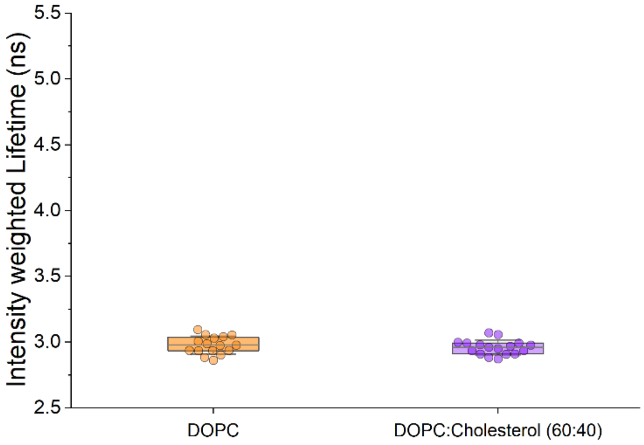

Figure S2. Mean intensity weighted lifetimes of Flipper-TR in eDICE GUVs made of lipid mixtures containing 100 mol% DOPC (orange) and DOPC:cholesterol (60:40 mol%) (purple). Box percentile 5-95, line in box is the mean and whiskers the SD. Each data point represents the average lifetime value of one individual GUV.

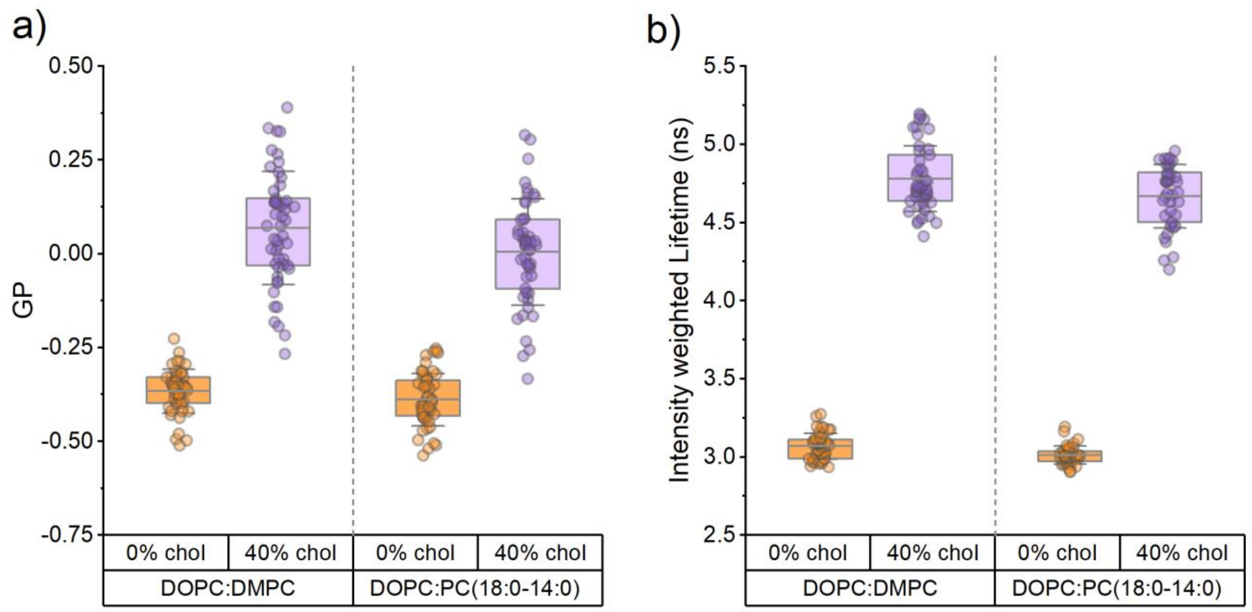

Figure S3. NR12A GP values (a) and Flipper-TR fluorescence lifetimes (b) of gel-swollen GUVs used for calibration. In each case, a comparison is shown between GUVs made of binary lipid mixtures without cholesterol (labelled as 0% chol) versus ternary lipid mixtures with cholesterol (labelled as 40% chol). The binary lipid compositions are DOPC:DMPC (60:40) and DOPC: PC(18:0-14:0) (60:40). The ternary lipid mixtures are DOPC:DMPC:cholesterol (36: 24: 40) and DOPC: PC(18:0-14:0): cholesterol (36: 24: 40). Box represents percentile 5-95, line in box is the mean, and whiskers the SD. Points are individual GUVs. The GUVs made of binary lipid mixtures show similar NR12A GP values and Flipper-TR fluorescence lifetimes as compared to those observed in eDICE GUVs with the same lipid composition. The NR12A GP values and Flipper-TR fluorescence lifetimes of the GUVs with a ternary lipid mixtures are comparable to the values observed in the binary eDICE GUVs after incubation with 100μM cholesterol (MβCD-CL)

Supplementary Video 1. Fluorescence confocal microscopy time-lapse showing phase separation of an eDICE GUV with an initial lipid composition of DOPC:DPPC (60:40 mol%) upon incubation with 100μM cholesterol (MβCD-CL) in the outer medium at room temperature. The membrane is labelled with Flipper-TR, the excitation wavelength is 488 nm and the emission is collected over a wavelength range between 575 nm and 635 nm. The recording is taken at the equatorial plane of the GUV.
